# Metagenome-assembled genomes from biological soil crusts in sandy sediments of Kitty Todd Nature Preserve, OH, USA

**DOI:** 10.1128/mra.00380-26

**Published:** 2026-05-29

**Authors:** Elena L. Peredo, Rachel Kulp, Fernando Rodriguez, Michael N. Weintraub, Maya Anand, Skye Bixler, Joshua Koller, Crystal Lee, Diya Mathai, Serena Tuytschaevers, Girish Kumar

**Affiliations:** 1 Thomas H. Gosnell School of Life Sciences, College of Science, Rochester Institute of Technology 6925, Rochester, New York, USA; 2 Marine Biological Laboratory, The Ecosystems Center 541653 , Woods Hole, Massachusetts, USA; 3 Department of Environmental Sciences, University of Toledo 7923, Toledo, Ohio, USA,; Montana State University, Bozeman, Montana, USA

**Keywords:** biological soil crusts, archaea, nitrogen metabolism

## Abstract

Biological soil crusts (BSCs) are complex structures composed of prokaryotes, green microalgae, fungi, and small mosses that bind soil particles together. To further understand the microbial composition and interactions among members of these consortia, we investigated the microbial diversity of BSCs found in a xeric patch in northwestern Ohio.

## ANNOUNCEMENT

Biological soil crusts (BSCs) are ecosystem engineers critical for soil stabilization, nutrient and organic carbon retention, water storage, and microhabitat creation. Many of the microorganisms in these crusts are diazotrophic and photosynthetic (1); in temperate forests, they also contribute to phosphorus cycling (2). This living soil layer is a well-characterized component of ecosystems in drylands, the Arctic, and alpine environments. However, despite their role in nutrient cycling, temperate BSCs remain understudied when compared to those in dry areas (2).

In July 2024, moss-dominated BSCs were collected from seven sites along a transect in a sand belt near the Oak Openings at Kitty Todd Nature Preserve near Toledo, Ohio, USA (41.620825–83.791731 to 41.622198–83.791421). Each site was separated by approximately 200 m. At every site, three replicate cores were collected less than 40 cm apart using sterile 50 mL Falcon tubes. Each core sampled an area of approximately 7 cm² in diameter. Samples were immediately transported to the lab and stored at −80°C until use. DNA was extracted from each of the three replicates at each site, and the resulting libraries were co-assembled by site.

A total of 0.25 g of each sample was randomly selected for DNA extraction using the DNeasy PowerSoil Pro Kit (Qiagen, Hilden, Germany) following the manufacturer’s instructions. DNA was quantified using a NanoDrop One (Thermo Fisher Scientific, Waltham, Massachusetts) and a Qubit 4.0 (Thermo Fisher Scientific). Metagenomic libraries were prepared with the Nextera XT Kit (Illumina, San Diego, California) and quantified using a TapeStation 4200 (Agilent Technologies, Santa Clara, California) prior to pooling. Libraries were size-selected to 300–600 bp using a PippinHT (Sage Science, Beverly, Massachusetts). Sequencing was performed on a NovaSeq 6000 (Illumina) at the Genomics Enterprise Center at the Rochester Institute of Technology (Rochester, NY, USA) using the standard workflow mode for paired-end sequencing and 250 cycles. Read quality was evaluated with FastQC v0.12.1 (3). A total of 1,247,841,772 reads (2 × 250 bp) were generated for an average of ~3 × 10^7^ reads per replicate, of which 95% were retained after trimming (1,187,535,424) with Trimmomatic v0.39 (4) using the following parameters: ILLUMINACLIP: TruSeq3-PE.fa 2:30:10 HEADCROP:19 LEADING:3 TRAILING:3 SLIDINGWINDOW:4:15 MINLEN:36. Co-assemblies included all three replicates from each site were generated with MEGAHIT v1.2.9 (5) and assessed using metaQUAST v5.2.0 (6). Contigs were binned into MAGs using MaxBin2 v3.0.2 (7), CONCOCT v1.1.0 (8), and MetaBAT2 v2.15 (9). All programs were run using default parameters unless otherwise specified.

**Fig 1:**
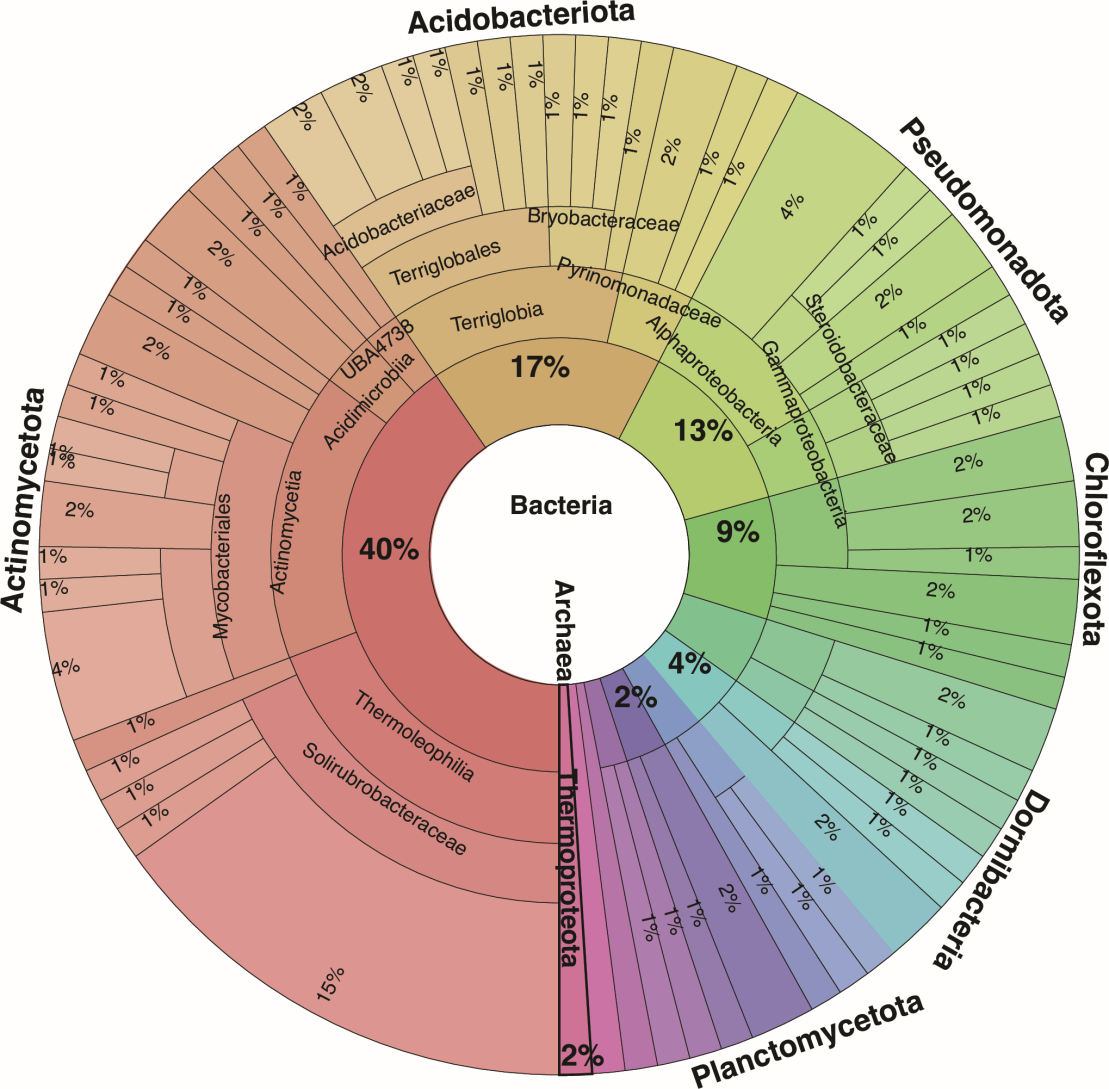
Taxonomic distribution of MAGs isolated from biological soil crusts in sand barren soils in Ohio. Taxonomy was assigned using GTDB-Tk and visualized using Krona, with percentages in the inner ring indicating phylum-level abundances.

The resulting set of 3,677 candidate bins was evaluated with QUAST (10), BUSCO v5.4.3 (11), and CheckM v1.2.0 (12) as implemented in the nf-core/mag v 4.0.0. pipeline (13). Of these, 202 bins met high-quality based on conserved marker genes (CheckM ≥ 90% completion, <10% contamination; BUSCO > 90%; Table 1) and were retained as MAGs. Taxonomic placement was calculated using GTDB-Tk (14) as implemented in Kbase (15). FastANI v1.3 (16) was used to calculate similarity. Redundant MAGs, that is sharing more than 99% ANI, were removed. After deduplication, a total of 101 MAGs were retained, corresponding to 2 archaeal and 99 bacterial, spanning over 21 classes (Fig. 1). MAGs were annotated with NCBI Prokaryotic Genome Annotation Pipeline (PGAP) (17). GC content ranged from 35% in Nitrososphaeraceae (Archaea) to 74% in Actinomycetospora. The average N50 across all MAGs was 21 Kb, ranging from 3 Kb to 145 Kb (Table 1). The smallest MAG corresponded to a *Sphingomicrobium*, with an estimated genome size of 2 Mb and approximately 2,300 genes, while the largest MAG was identified within Bryobacteraceae with a 9 Mb genome. Thirty-three MAGs had RED values <0.90 and 25 were identified as taxonomic novelties using combined RED values and placement of the genome in the reference tree as implemented in GTDB-Tk (Table 1), consistent with the expectations for an ecosystem underrepresented in current databases.

**TABLE 1:** Summary of statistics for the MAGs generated in this announcement

Accession Link	Completeness	Contamination	Genome size (bp)	Contigs	N50 contigs	Longest contig	GC%	Predicted genes	Coverage	Classification	MSA AA^*a*^ percent	RED^*b*^ value
JBVPXF000000000	96.4	3.99	4,956,861	369	23,844	136,987	66.3	5,063	48×	Acetobacteraceae	79.66	0.88
JBVPXG000000000	93.77	8.13	7,255,405	1,125	11,120	72,959	62.8	7,741	21×	Acetobacteraceae	81.17	0.97
JBVPUP000000000	96.3	2.31	4,040,109	264	33,123	117,042	67.8	4,067	54×	Acidimicrobiales	90.74	0.75
JBVPUQ000000000	98.01	5.94	3,282,648	148	36,431	95,982	69.5	3,407	103×	Acidimicrobiales	87.13	0.75
JBVPUR000000000	91.45	3.85	3,897,580	546	9,705	32,892	66.9	4,342	17×	Acidimicrobiia	83.16	0.86
JBVPUG000000000	96.34	2.8	3,467,168	133	50,575	132,196	62	3,088	37×	Acidobacteriaceae	87.45	0.93
JBVPUH000000000	90.61	6.93	4,615,874	573	13,051	81,496	58.4	4,289	12×	Acidobacteriaceae	76.88	0.88
JBVPUI000000000	90.23	8.76	6,652,020	944	11,529	75,704	58.9	5,832	24×	Acidobacteriaceae	67.17	0.95
JBVPUJ000000000	92.39	3.66	8,767,866	1,381	10,973	80,404	58.3	7,997	18×	Acidobacteriaceae	76.66	0.96
JBVPVA000000000	93.96	8.82	6,020,445	1,212	6,734	39,580	74.4	6,502	54×	Actinomycetospora sp.	75.63	0.98
JBVPWB000000000	95.73	3.87	2,665,929	244	16,331	94,254	63.6	2,831	27×	Actinomycetota	90.86	0.77
JBVPWC000000000	97.44	4.27	3,341,125	394	11,444	58,698	70.2	3,397	32×	Actinomycetota	87.53	0.66
JBVPXI000000000	98.45	5.44	5,590,372	295	28,998	135,010	63.3	5,460	38×	Bradyrhizobium sp.	85.62	0.95
JBVPXJ000000000	94.41	9.58	6,992,952	923	12,085	66,499	64.3	7,152	25×	Bradyrhizobium sp.	72.85	1.00
JBVPXK000000000	97.28	4.51	5,202,923	426	18,378	83,802	63.8	5,345	21×	Bradyrhizobium sp.	91.74	0.95
JBVPXL000000000	97.09	8.4	7,083,908	589	19,009	106,607	64	7,117	33×	Bradyrhizobium sp.	78.67	1.00
JBVPUD000000000	92.67	4.06	8,343,938	1,897	6,617	38,299	57.3	8,673	20×	Bryobacteraceae	71.5	0.86
JBVPUE000000000	96.27	5.92	9,283,950	1,336	12,554	76,545	54.7	8,845	17×	Bryobacteraceae	85.04	0.98
JBVPUF000000000	95.96	4.2	5,423,377	838	9,189	47,434	56.4	5,304	16×	Bryobacteraceae	82.94	0.91
JBVPUC000000000	94.66	4.33	4,748,924	375	24,762	107,030	54.4	4,177	17×	Candidatus Acidiferrum sp.	84.11	0.93
JBVPUM000000000	98.08	3.85	5,910,239	175	52,259	257,313	55.3	4,947	34×	Candidatus Angelobacter sp.	91.86	0.94
JBVPWQ000000000	93.43	7.34	2,314,772	431	7,025	34,114	67.1	2,686	16×	Candidatus Dormibacteraceae	72.73	0.71
JBVPWR000000000	93.83	0.93	2,141,315	99	40,145	108,308	67.3	2,306	57×	Candidatus Dormibacteraceae	89.02	0.70
JBVPWS000000000	95.68	3.99	4,257,725	695	8,389	38,475	73.3	4,486	25×	Candidatus Dormibacteria	89.33	0.94
JBVPWT000000000	91.51	7.88	2,524,800	289	16,898	53,760	69.4	2,571	60×	Candidatus Dormibacteria	82.11	0.96
JBVPWV000000000	90.16	5.48	4,177,112	598	10,019	57,960	70.7	4,269	187×	Candidatus Elarobacter sp.	75.73	0.96
JBVPWW000000000	94.4	9.26	5,046,313	280	30,449	154,222	70.1	5,075	46×	Candidatus Elarobacter sp.	77.16	0.95
JBVPWY000000000	91.96	6.02	2,537,398	322	11,182	40,531	63.8	2,643	14×	Candidatus Eremiobacter sp.	69.95	0.96
JBVPWX000000000	90.31	1.95	2,431,513	317	11,424	42,135	65	2,540	15×	Candidatus Eremiobacteraceae	75.89	0.83
JBVPUN000000000	96.58	8.41	5,304,348	1,127	6,637	104,238	60.2	5,314	71×	Candidatus Korobacteraceae	83.06	0.90
JBVPWD000000000	96.3	0.08	4,132,019	225	41,883	164,702	58.4	3,843	22×	Capsulimonadaceae	86.97	0.80
JBVPWE000000000	96.91	0.21	3,659,389	371	14,643	64,051	62.2	3,836	24×	Capsulimonadaceae	89.2	0.87
JBVPWF000000000	93.52	6.14	4,010,803	654	9,180	41,483	61.4	4,304	19×	Capsulimonadaceae	81.05	0.88
JBVPXP000000000	95.68	3.35	4,602,392	585	10,880	42,119	65.2	4,493	21×	Casimicrobiaceae	87.51	0.90
JBVPXH000000000	94.2	1.35	2,966,617	228	19,456	67,198	68.5	3,027	24×	Caulobacteraceae	93.74	0.96
JBVPWN000000000	97.69	1.19	4,895,287	291	38,082	145,150	65.3	4,526	137×	Chloroflexota	89.51	0.83
JBVPWO000000000	100	3.47	4,148,850	229	31,962	144,251	60.6	4,033	30×	Chloroflexota	89.37	0.83
JBVPWP000000000	96.86	4.21	7,138,756	525	25,396	129,040	68	6,952	30×	Chloroflexota	86.85	0.95
JBVPXS000000000	94.03	4.84	3,121,120	541	7,843	41,030	58.1	3,110	17×	Chthoniobacterales	77.84	0.93
JBVPWK000000000	96.08	6.93	7,574,296	710	16,732	81,008	51.1	6,897	17×	Dictyobacter sp.	80.42	0.93
JBVPWL000000000	97.19	5.81	7,925,781	643	19,392	99,522	51.6	7,200	27×	Dictyobacter sp.	85.14	0.93
JBVPVI000000000	91.65	6.25	3,892,774	501	10,863	38,158	72.8	3,756	50×	Gaiellales	80.38	0.65
JBVPWZ000000000	97.8	7.99	4,966,267	648	11,684	111,338	68.7	4,557	36×	Gemmatimonadaceae	91.2	0.88
JBVPXA000000000	93.31	9.04	3,910,829	428	14,232	61,175	68.3	3,731	22×	Gemmatimonadaceae	79.84	0.94
JBVPXB000000000	93.88	3.31	2,823,658	311	12,710	39,486	63.1	2,759	19×	Gemmatimonadales	83.1	0.92
JBVPWH000000000	96.97	3.59	5,916,010	599	14,697	62,684	57.2	5,758	20×	Herpetosiphonaceae	85.42	0.98
JBVPWG000000000	98.77	0.44	4,092,002	65	145,054	362,727	41.9	3,570	33×	Ilyomonas sp.	96.54	0.95
JBVPXE000000000	90.87	9.17	7,227,456	1,907	5,335	29,079	65.3	6,949	13×	Isosphaeraceae	61.81	0.85
JBVPUT000000000	93.35	4.08	3,651,035	146	41,873	121,075	70.5	3,730	24×	Jatrophihabitans sp.	79.21	0.93
JBVPUU000000000	95.05	5.49	4,573,566	438	22,677	158,398	66.5	4,823	18×	Jatrophihabitantaceae	88.28	0.91
JBVPWI000000000	96.97	5.04	6,034,915	885	10,729	51,204	48.7	5,753	19×	Ktedonobacteraceae	84.17	0.87
JBVPWJ000000000	93.23	7.02	5,933,713	820	9,864	45,062	52.9	5,189	28×	Ktedonobacteraceae	83.77	0.88
JBVPWM000000000	99.17	7.15	7,119,265	394	28,345	156,782	49.2	6,057	80×	Ktedonobacteraceae	88.38	0.98
JBVPUS000000000	91.38	5.72	3,695,483	344	16,547	68,848	70.4	3,775	87×	Lapillicoccus sp.	83.1	0.88
JBVPUW000000000	97.91	7.42	5,931,477	561	15,833	60,651	68.7	6,114	28×	Mycobacterium sp.	79.94	1.00
JBVPUX000000000	97.36	5.99	4,439,930	550	11,597	70,027	66.3	4,689	39×	Mycobacterium sp.	80.4	0.99
JBVPXC000000000	94.82	7.59	4,594,449	742	8,160	48,017	69.3	4,826	16×	Myxococcales	82.8	0.93
JBVPUY000000000	97	1.98	4,596,173	291	23,849	116,746	70.5	4,271	23×	Nakamurella sp.	86.67	0.95
JBVPTW000000000	96.37	9.79	2,762,220	1,035	3,102	16,544	37.7	3,184	32×	Nitrososphaeraceae archaeon	75.08	0.95
JBVPTX000000000	98.54	1.94	2,922,502	592	6,437	37,355	35.6	3,229	14×	Nitrososphaeraceae archaeon	89.15	0.95
JBVPVF000000000	94.65	3.39	3,223,235	175	32,240	153,717	70.1	3,228	55×	Nocardioidaceae	91.76	0.81
JBVPVG000000000	94.78	6.1	4,092,481	412	15,678	64,476	67.5	4,209	21×	Nocardioidaceae	79.07	0.87
JBVPUK000000000	95.64	5.96	5,530,623	594	18,380	110,299	62.3	4,727	25×	Occallatibacter sp.	86.85	0.97
JBVPUL000000000	96.98	2.69	7,072,328	639	17,548	84,255	60.4	6,096	28×	Occallatibacter sp.	90.59	0.97
JBVPXO000000000	90.15	7.43	7,248,414	1,669	5,277	25,175	61.8	7,565	11×	Paraburkholderia caledonica	69.71	-
JBVPUV000000000	97.5	1.58	6,427,346	597	16,296	63,005	68.7	5,876	20×	Planosporangium sp.	87.49	0.91
JBVPUZ000000000	92.39	8.97	6,137,193	1,241	6,282	34,543	66.5	7,160	30×	Pseudonocardiaceae	78.49	0.91
JBVPVB000000000	92.17	7.53	5,088,090	590	14,653	66,639	66.3	5,230	146×	Pseudonocardiaceae	89.06	0.99
JBVPVC000000000	89.23	3.81	5,372,936	606	16,424	72,996	66.7	5,516	38×	Pseudonocardiaceae	89.79	0.95
JBVPVD000000000	94	7.24	5,986,343	655	13,678	87,513	66.7	6,304	19×	Pseudonocardiaceae	86.38	0.95
JBVPVE000000000	89.39	7.5	4,916,738	745	9,020	45,724	65.7	5,212	19×	Pseudonocardiaceae	80.62	0.93
JBVPTY000000000	91.83	7.31	5,560,137	466	17,882	58,818	59.7	4,831	13×	Pyrinomonadaceae	84.13	0.88
JBVPTZ000000000	93.23	8.87	4,323,336	1,393	3,859	26,775	62	4,523	12×	Pyrinomonadaceae	77.7	0.95
JBVPUA000000000	91.45	9.95	5,328,188	135	69,614	195,839	62.6	4,482	43×	Pyrinomonadaceae	89.14	0.95
JBVPUB000000000	89.32	6.99	4,707,391	147	57,438	237,874	62.3	3,977	35×	Pyrinomonadaceae	81.55	0.94
JBVPVJ000000000	93.16	2.31	3,055,699	187	28,900	159,278	69.6	3,075	61×	Solirubrobacteraceae	87.07	0.83
JBVPVK000000000	90.94	4.7	3,435,890	140	57,464	142,446	68	3,529	55×	Solirubrobacteraceae	83.42	0.96
JBVPVL000000000	99.15	7.26	3,728,137	260	24,947	126,571	68.1	3,758	129×	Solirubrobacteraceae	88.94	0.89
JBVPVM000000000	91.97	7.07	2,660,156	400	10,965	46,478	69.2	2,903	29×	Solirubrobacteraceae	79.17	0.89
JBVPVN000000000	91.03	3.08	3,876,704	297	24,955	101,342	68	3,950	42×	Solirubrobacteraceae	82.42	0.90
JBVPVO000000000	89.09	5.1	5,617,645	709	12,415	76,718	69.1	6,028	40×	Solirubrobacteraceae	74.7	0.97
JBVPVP000000000	92.74	4.9	3,339,434	195	28,131	101,839	66.6	3,481	33×	Solirubrobacteraceae	81.87	0.91
JBVPVQ000000000	94.44	4.27	3,103,373	257	19,076	76,173	66.9	3,119	28×	Solirubrobacteraceae	82.13	0.91
JBVPVR000000000	97.44	8.63	3,469,725	191	30,482	155,864	67.8	3,562	50×	Solirubrobacteraceae	82.28	0.91
JBVPVS000000000	94.16	2.65	3,210,338	291	17,758	67,131	70.1	3,404	22×	Solirubrobacteraceae	85.36	0.85
JBVPVT000000000	90.47	4.56	4,839,309	679	9,852	49,679	69.8	5,142	21×	Solirubrobacteraceae	72.35	0.92
JBVPVU000000000	92.96	2.85	2,787,999	432	9,360	53,846	67	3,002	31×	Solirubrobacteraceae	82.46	0.95
JBVPVV000000000	94.44	2.78	2,901,046	215	21,392	52,389	71	2,944	38×	Solirubrobacteraceae	84.73	0.91
JBVPVW000000000	95.13	3.85	5,281,434	366	21,624	83,806	70.9	5,271	64×	Solirubrobacteraceae	84.05	0.92
JBVPVX000000000	94.03	4.71	3,584,332	482	10,276	45,416	66.2	3,836	13×	Solirubrobacteraceae	81.39	0.96
JBVPVY000000000	89.16	2.26	3,646,960	501	10,382	47,219	70.3	3,883	17×	Solirubrobacteraceae	73.33	0.96
JBVPVZ000000000	95.81	5.41	2,797,434	140	33,916	96,019	70.3	2,894	50×	Solirubrobacteraceae	86.61	0.90
JBVPWA000000000	95.94	5.36	3,054,394	69	79,612	195,219	64.6	2,746	53×	Solirubrobacteraceae	86.45	0.97
JBVPXM000000000	94.9	8.92	3,087,973	394	13,119	56,002	64	3,359	19×	Sphingomicrobium sp.	82.2	0.97
JBVPXN000000000	89.98	4.2	2,079,431	255	11,194	44,855	64.3	2,285	20×	Sphingomicrobium sp.	80.2	0.97
JBVPXQ000000000	91.07	5.08	3,789,810	537	9,445	47,432	68.5	3,751	13×	Steroidobacteraceae	86.77	0.91
JBVPXR000000000	97.46	4.74	4,652,572	145	58,123	221,463	62.2	4,271	34×	Steroidobacteraceae	97.52	0.94
JBVPVH000000000	92.24	8.91	7,621,928	1,361	7,394	39,381	71.9	8,006	30×	Streptosporangiaceae	77.52	0.98
JBVPXD000000000	90.04	5.11	6,686,751	1,628	4,806	90,525	68.9	6,025	34×	Tepidisphaeraceae	70.33	0.82
JBVPUO000000000	99.57	3.3	3,586,718	370	16,461	66,439	53.6	3,388	15×	Terriglobales	90.01	0.97
JBVPWU000000000	95.37	1.73	2,667,775	211	29,205	110,408	57.7	2,757	17×	Vulcanimicrobiaceae	87.65	0.75

^*a*^ Multiple Sequence Alignment (MSA) of amino acid (AA) sequences.

^*b*^ (RED) Relative Evolutionary Divergence. Taxonomical novelty are indicated by underlining.

## Data Availability

This Whole Genome Shotgun project has been deposited in GenBank under the accession PRJ1305458. SRA data can be found as accessions SSR34988617–SSR34988637 and the MAGs as accessions JBVPTW000000000–JBVPXS000000000 (see Table 1 for specific accession numbers).
